# Family-Centered Care in Juvenile Justice Institutions: A Mixed Methods Study Protocol

**DOI:** 10.2196/resprot.5938

**Published:** 2016-09-12

**Authors:** Inge Simons, Eva Mulder, Henk Rigter, René Breuk, Wander van der Vaart, Robert Vermeiren

**Affiliations:** ^1^ Curium, Leiden University Medical Center Leiden Netherlands; ^2^ Intermetzo Zutphen Netherlands; ^3^ University of Humanistic Studies Utrecht Netherlands

**Keywords:** adolescents, delinquency, juvenile offenders, family-centered care

## Abstract

**Background:**

Treatment and rehabilitation interventions in juvenile justice institutions aim to prevent criminal reoffending by adolescents and to enhance their prospects of successful social reintegration. There is evidence that these goals are best achieved when the institution adopts a family-centered approach, involving the parents of the adolescents. The Academic Workplace Forensic Care for Youth has developed two programs for family-centered care for youth detained in groups for short-term and long-term stay, respectively.

**Objective:**

The overall aim of our study is to evaluate the family-centered care program in the first two years after the first steps of its implementation in short-term stay groups of two juvenile justice institutions in the Netherlands. The current paper discusses our study design.

**Methods:**

Based on a quantitative pilot study, we opted for a study with an explanatory sequential mixed methods design. This pilot is considered the first stage of our study. The second stage of our study includes concurrent quantitative and qualitative approaches. The quantitative part of our study is a pre-post quasi-experimental comparison of family-centered care with usual care in short-term stay groups. The qualitative part of our study involves in-depth interviews with adolescents, parents, and group workers to elaborate on the preceding quantitative pilot study and to help interpret the outcomes of the quasi-experimental quantitative part of the study.

**Results:**

We believe that our study will result in the following findings. In the quantitative comparison of usual care with family-centered care, we assume that in the latter group, parents will be more involved with their child and with the institution, and that parents and adolescents will be more motivated to take part in therapy. In addition, we expect family-centered care to improve family interactions, to decrease parenting stress, and to reduce problem behavior among the adolescents. Finally, we assume that adolescents, parents, and the staff of the institutions will be more satisfied with family-centered care than with usual care. In the qualitative part of our study, we will identify the needs and expectations in family-centered care as well as factors influencing parental participation. Insight in these factors will help to further improve our program of family-centered care and its implementation in practice. Our study results will be published over the coming years.

**Conclusions:**

A juvenile justice institution is a difficult setting to evaluate care programs. A combination of practice-based research methods is needed to address all major implementation issues. The study described here takes on the challenge by means of practice-based research. We expect the results of our study to contribute to the improvement of care for adolescents detained in juvenile justice institutions, and for their families.

## Introduction

Delinquent youths often come from malfunctioning families. The problems of these families vary from disturbed mutual relationships, to drug abuse, delinquency, and poor mental health among family members [1,2]. In adolescents, the risk of committing criminal offenses is related to family factors such as poor parenting skills, lack of emotional support from parents, neglect and physical abuse, and criminal behavior of family members [3]. Family therapy reduces criminal behavior of adolescents [4], and also improves family functioning [5-7]. Therefore, intervention programs for delinquent adolescents should focus not only on the youth but also on the family in order to have the adolescent abstain from criminal activities [3,8-10]. Such family-centered intervention programs could include family therapy [11].

Whereas family problems are related to youth delinquency, the protective effects of positive parenting should not be ignored [10]. Involving parents during their child’s detention is important for improved outcomes for youth [12]. Parental engagement and emotional support help to improve outcomes for youth in terms of treatment engagement, well-being, behavior, and recidivism [10,13]. Additionally, recidivism rates decline if parents are more involved with their children in juvenile court [14].

Until the start of the project that led to the current paper, care in youth detention centers in the Netherlands, called juvenile justice institutions (JJIs), has been mainly youth-focused, with little attention for the family. Realizing the importance of family factors, the Netherlands Government decided to encourage JJIs to adopt a family-centered approach. This has resulted in incorporating a few family-centered actions in all JJIs’ usual care (UC) programs, such as staff calling parents once a week or inviting parents to key meetings where the intervention plan for their child is being discussed [15]. However, JJIs were found to not properly adhere to this rather modest way of involving parents [16], and methods to involve parents have not been systematically implemented in practice [17]. The need for programs stimulating family involvement during a child’s detention is not only of concern in the Netherlands, but is internationally recognized [18,19]. Families need to be heard, empowered, supported, and the ties between adolescents and their parents need to be strengthened by improving communication [18].

Previous studies have elaborated on the challenges to involve parents in juvenile justice services. Characteristics from parents and from the juvenile justice system can negatively influence parental involvement [12,14]. These parent characteristics include lack of resources for transportation, time constraints, fear of losing a job because of the time-consuming process, competing demands, and lack of child care for other children. Also, there may be medical concerns, and parents may feel failed and tired after years of struggle with their child’s problem behavior. Parents may mistrust the institution because of previous negative experiences with service providers. Characteristics of the justice system that could hamper parental involvement include staff’s lack of respect towards parents, their unwillingness to work with parents, confusing communication with parents, time-consuming and not family-friendly processes, the lack of a cultural competent system, and the lack of communication in parents’ native language [12,14]. Additionally, staff’s negative attitudes can give parents the impression that they are seen as the problem instead of part of the solution [14]. Other factors are able to both facilitate and hinder parental involvement, such as availability of staff and flexibility of the system [12]. A positive relationship between parents and their child prior to detention can positively influence parental engagement during their child’s detention [20].

Dissatisfied with the underdeveloped level of family-centered care in the Netherlands, two JJIs participated in the Academic Workplace Forensic Care for Youth (AWFZJ) to develop and evaluate a program for family-centered care (FC) [21]. The AWFZJ is a practice-based research collaboration between two JJIs, two universities, two colleges of applied sciences, and two centers for child and adolescent psychiatry. The AWFZJ developed two versions of the FC program, one for youth detained in short-term stay groups and one for youth detained in long-term stay groups.

We decided to examine if FC is beneficial for detained youths and their parents. We report here on the design of a study to evaluate FC in the first two years after the first steps of its implementation in short-term stay groups. Each short-term stay group has room for 10 adolescents. The groups are supported and monitored by JJI staff, so-called group workers (mostly social workers). The aim of the current paper is to describe the study protocol and to stress the potential of research studies in a challenging setting such as a JJI with its ethical dilemmas, the unfamiliarity of staff with research methodology, and with a difficult population with low treatment motivation [22-24].

## Methods

### Design

Our study has a practice-based nature. Carrying out research in a setting such as a JJI is challenging, as it is in most practice-based studies [25]. It is virtually impossible to organize a randomized controlled trial in a JJI. First, judges are not likely to agree with randomizing adjudicated adolescents to different detention conditions. Second, JJIs struggle with relative instability of staff due to high turnover and high rates of absenteeism [26]. Another barrier for conducting research in JJIs is the unfamiliarity among most of the institution’s staff with the principles and benefits of research studies [23]. To prepare JJI personnel for implementing and evaluating FC, we trained them to internalize FC rationale and FC practice and we organized a seven-month pilot stage. In the remainder of the pilot stage, we found FC short-term stay groups to differ in number and nature of family-oriented actions, although all group workers had received the same training. Also, we noticed that not every parent visited their child or attended every kind of family activity organized by the JJI. Additionally, the preliminary analyses of the pilot data showed the surprising finding that most parents and youths report few family problems, while at the same time they report motivation for family therapy. In setting up the actual study, we used feedback from staff and the results of monitoring the groups during the pilot stage to improve the FC program.

**Figure 1:**
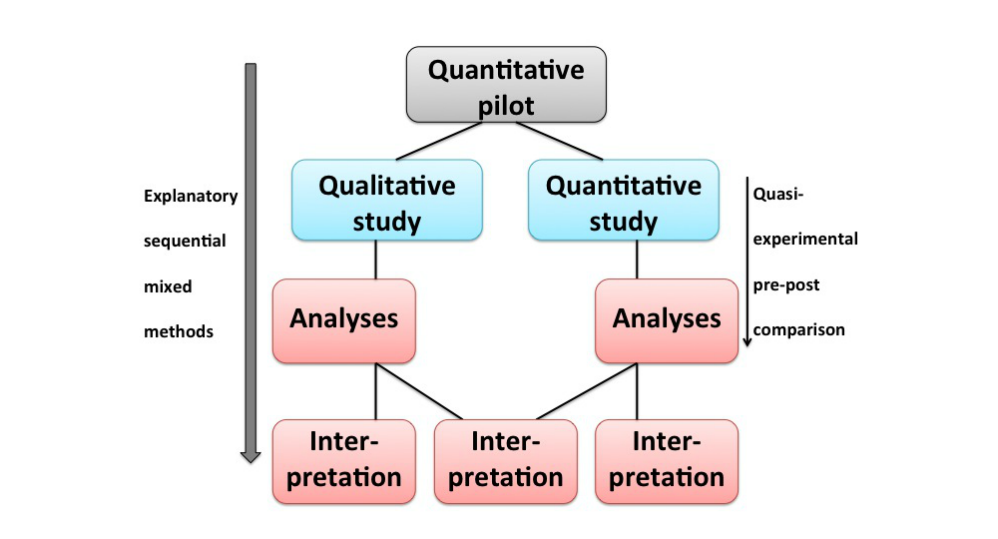
Study design.

Evaluating the pilot stage gave rise to our final study design, in which the pilot is considered as the first stage, see Figure 1.

In our study, we employ a mixed methods design in which quantitative and qualitative research methods are combined [27]. In mixed methods studies, qualitative and quantitative stages of data collection can occur concurrently or sequentially and can be nested in each other [28,29]. We utilize an explanatory sequential mixed methods design [30] with a large concurrent stage. The first stage of the sequence consists of the quantitative pilot. The second stage of the sequence involves concurrent qualitative and quantitative components. In the third stage, which is integral part of the study, we distinguish data analyses and interpretation. Part of the interpretation concerns the integration of qualitative and quantitative outcomes.

The qualitative part of our study is used to elaborate on the preceding quantitative pilot outcomes and to discuss further interpretations of the quantitative quasi-experimental pre-post study outcomes. This qualitative part can help to gain insight into underlying mechanisms influencing parent participation and is therefore considered explanatory [31]. Understanding these mechanisms can contribute to overcoming possible obstacles in organizing family-oriented activities and can therefore improve FC.

The quantitative part in the second stage of our study will be carried out parallel to the qualitative part. This quantitative part is a pre-post comparison of two programs–FC and UC–for adolescents placed in short-term stay groups of two JJIs. This comparison is quasi-experimental, as no randomization will take place in assigning youth to either a FC or a UC group.

The details about the stages and the contents of our study were discussed and detailed in workgroups of JJI staff and research staff, in an attempt to render FC study activities attainable in daily practice and to prepare staff for the requirements of our study. Over the course of our study, we will regularly discuss the study’s progress and its practical impact on staff in these workgroups. Additionally, registered information of staff’s family-oriented actions will be shared during team meetings, which offers insight into the success of implementing FC and its program integrity. This feedback can stimulate family-centered activities. These overviews will also be provided on a regular basis to the managements of the two JJIs, enabling them to monitor and direct the organization of family-centered activities in the institutions as outlines in the program manual.

### Study Objectives and Research Questions

The overall aim of our study is to evaluate FC in the first two years after the first steps of its implementation in short-term stay groups in JJIs. The key question to be answered in the quantitative part in the second stage of the study is if FC has additional value compared to UC. We will test the following hypotheses comparing FC with UC during detention: (1) FC increases parents’ involvement with their detained child; (2) FC increases the motivation of the adolescent and his parents for accepting treatment and guidance by JJI staff and for taking part in family meetings; (3) FC adolescents show less problem behavior; (4) FC improves family interactions; (5) FC parents experience less parenting stress; (6) FC youth more often return to their families’ home upon discharge; (7) FC enhances adolescents’ and parents’ satisfaction with the JJI; (8) In FC groups, JJI staff members are more satisfied, feel more confident in their contact with parents, and more often incorporate the family perspective in their thinking.

Finally, we will study if parents who participate in family-centered activities, differ from parents who do not participate based on characteristics such as proximity to the JJI, age of their child, duration of his stay, and baseline outcomes in other demographics, family functioning, parenting stress, treatment motivation, and satisfaction.

The aim of the qualitative part of the study is to trace which factors influence parental involvement. We will interview adolescents, parents, and group workers from short-term stay groups based on the following research questions: (1) How do adolescents, parents, and group workers feel about the current involvement of parents in FC and UC? (2) What are the attitudes of FC and UC group workers towards working with parents? (3) What are the needs, wishes, and expectations of adolescents, parents, and group workers concerning FC?

### Setting

This study will be carried out in two JJIs in the Netherlands. A juvenile judge can refer an adolescent to a short-term stay group in a JJI for pre-trial detention. Depending on the interim ruling of the juvenile judge, the time spent in pre-trial detention can last for a few days up to a maximum of customarily 90 days. As a rule, the juvenile judge refers the adolescent to a JJI close to the home of the youth. The JJI’s secretarial office monitors a group’s capacity and decides on which group the adolescent is placed.

One of the JJIs has three short-term stay groups. The management of this institution chose two of these groups for a step-by-step implementation of the FC program, while the third group will continue to offer UC. Of the two short-term stay groups in the other JJI, the management chose one to offer FC, and the other UC. The managements of the two JJIs based their choices for the groups starting with the implementation of FC on pragmatic considerations. Because the JJIs are required to fill free slots in the living groups if new adolescents are referred to the institutions, the assignment of adolescents to groups is not dependent on characteristics of youths and is therefore without bias.

Each team of about 10 group workers is headed by a team leader and collaborates with a psychologist or pedagogue (hereafter jointly referred to as psychologist), who is responsible for coordinating the treatment the adolescent will receive.

### Participants

#### Adolescents and Their Parents

All adolescents in our study will be boys, as girls are not referred to the two JJIs concerned. The boys will be between 12 and 18 years old at the time of placement. All youth placed in a FC group will be offered FC, but not all of them will be included in our study. An adolescent will be excluded (1) if his stay in the short-term stay group lasts less than 14 days (we need a minimum of two weeks to complete all assessments for the study); (2) if he does not have a parent or a parent figure; (3) if he already participated in our study during a previous stay; (4) if he does not understand Dutch; (5) if he and his parents refuse to take part in the assessments; (6) if he is already sentenced by the juvenile judge to a so-called PIJ order (Placement in an Institution for Juveniles for mandatory treatment) which implies long-term detention with treatment, or (7) if he is temporarily transferred from another institution.

As our assessments will be part of the Routine Outcome Monitoring (ROM) and of the standard screening and diagnostic procedures, psychologists can withhold the adolescent or his family from assessments, for example in case of severe psychiatric disorders. Reasons for excluding participants from the study will be noted. Consequently, we will first consult psychologists before approaching adolescents and their parents for the interviews. In general, following the psychologists’ advice, we will not approach them in case of an alleged sex crime or when severe psychiatric disorders such as mental retardation, psychosis, autism, or acute suicidal behaviors are present.

Because the questionnaires in the quantitative part of our study are embedded in the standard procedures in the institutions, no incentives will be used for youth and parents. For the interviews, however, youths will receive extra television time in their rooms and parents will receive a small incentive such as a mug filled with chocolates and a personal thank you note.

#### Staff

All staff allocated to the short-term stay groups in our study will be included in the quantitative part. In order to promote program integrity and to avoid contamination, group workers who work at the FC groups will preferably not work in the UC groups, and vice versa. The JJIs agreed to ensure as much staff-stability in the teams as possible, and to make an effort to keep staff consistent per group.

In addition, we will interview the group workers from the first two FC groups for the qualitative part of our study, as well as all group workers from the two UC groups. In each JJI, we will interview group workers from one FC and from one UC group.

At certain milestones during the study, we will bring a cake to the team meeting as an incentive for group workers for their family-centered activities or research-related activities. Team leaders will also discuss these activities in evaluation meetings with the group workers. For group workers’ participation with the interviews, they will receive the same incentive as parents.

### Recruitment and Sample Size

Adolescents and parents are informed of the JJI’s research activities by a flyer in the information leaflets from the JJI. The flyer informs that the data will be used anonymously in research studies and that parents can address their questions concerning these activities to their child’s mentor (one of the group workers) or to the psychologist.

The JJIs in the Netherlands jointly apply ROM and standard screening and diagnostic procedures for detained adolescents and their parents. As our assessments will be embedded in these procedures, the quantitative part of our study will use data collected in the two participating JJIs by these means. Recruitment of adolescents and their parents in the quantitative part of our study will last 21 months, including the pilot stage of 7 months. Based on records from 2011, the year prior to the pilot stage, we estimate that in 21 months, 300 adolescents will be placed in the groups concerned. Taking into account the exclusion criteria, we expect to recruit 160 adolescents and parents for the present study. Based on previous research, this number suffices for establishing statistically significant differences on quantitative measures between the two conditions [9].

As for qualitative studies, 10 interviews are generally sufficient to achieve saturation (ie, the point where additional interviews do not yield new essential information regarding the research question) [32]. Once an eligible adolescent is placed in a short-term stay group (either FC or UC), he and his parents will be invited to participate in the qualitative part of the study. If they are willing to participate, an appointment will be made for the interview. We will interview 10 boys (5 aged < 16 years and 5 aged > 16 years) in each JJI (N=20). We will also interview 20 parents (10 in in each JJI, 10 fathers and 10 mothers, 10 with a detained child aged < 16 years, and 10 with a detained child aged > 16 years). Finally, we will interview 20 FC group workers and 20 UC group workers.

### Programs

#### Family-Oriented Activities in Usual Care

According to the Dutch guidelines for UC, the adolescent’s mentor calls the parents within the first 10 days of placement of the youth to agree on weekly moments of telephone contact and to invite them for a meeting in the group, including a tour of the institution and its intramural school. The adolescent’s psychologist is invited to join part of that meeting as well. After the first 10 days, the mentor discusses which goals the adolescent wants to achieve and asks parents to sign for agreement. After three weeks, the mentor informs parents about the treatment plan and provides them with the opportunity to give feedback. Parents are invited for a meeting to discuss the second treatment plan after 12 weeks. If family-evenings are organized and if adolescents receive diplomas, parents are invited. Finally, parents may possibly be involved in treatment interventions for their child and in family therapy. All this is UC as outlined on paper; however, in practice these family-centered activities are barely translated into daily routine [16].

#### Family-Centered Care

An important aspect of FC is the training, ongoing coaching, and yearly booster sessions that JJI staff receive in working with parents. This training enables staff to adhere to the FC program with its more comprehensive and more structured family-oriented activities. In FC, staff members actively motivate parents to visit their detained child frequently and to take an interest in their child’s progress. Staff members also encourage parents to visit their child’s group and to join group activities such as cooking, sports, and playing games. The first phase of a youth’s detention is considered important in FC as the existing crisis is seen as an opportunity to establish engagement and build alliance with parents. A lot of emphasis is placed on the meeting in the third week of a child’s detention. During this meeting, the psychologist first meets the parents alone to learn about the family. Later, the adolescent and his mentor join the meeting. Parents are also invited for a variety of other meetings with staff, other parents, and youths where particular themes of general interest are being highlighted. Further, staff members actively and urgently invite parents to attend and have a say in all the meetings where the goals and the progress of the treatment plan for their child are being discussed. FC staff members are constantly in touch with the parents and give them regular (at least once a week) feedback on how their child is doing. If desired, parents can sign up for family therapy together with their child. This therapy–multidimensional family therapy (MDFT) or functional family therapy (FFT)–may already start when the adolescent is detained and will then be continued on an outpatient basis upon discharge of the adolescent from the JJI.

### Procedure and Instruments of the Quantitative Part of the Study

#### Assessments

**Figure 2:**
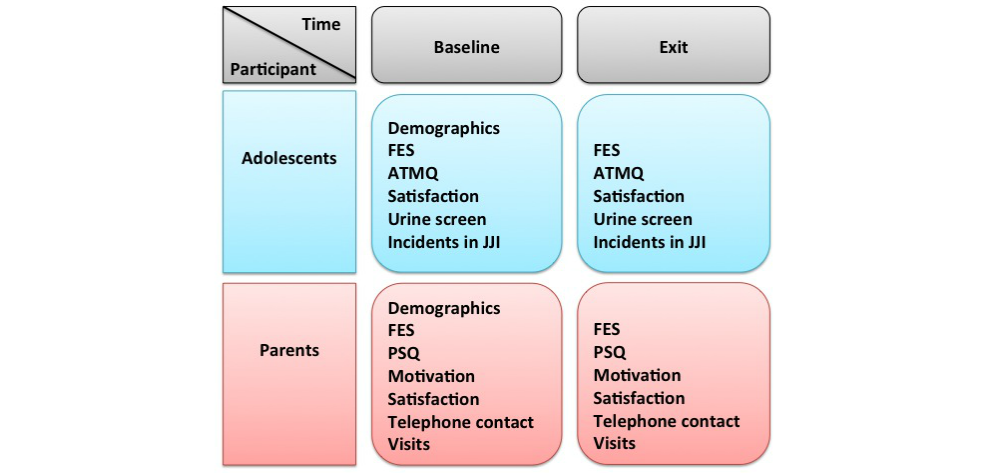
Overview of the quantitative measures for adolescents and parents; (FES) Family Environment Scale, (ATMQ) Adolescent Treatment Motivation Questionnaire, (JJI) Juvenile Justice Institution, (PSQ) Parenting Stress Questionnaire.

The baseline assessment for adolescents and parents will take place in the third week of detention. The second (exit) assessment will be held in the week of the adolescent’s departure from the short-term stay group. Although our assessments will be embedded in ROM and in the standard screening and diagnostic procedures of JJIs, we will assist in scheduling assessments and we will help to interpret the scores of family-oriented questionnaires so that they are usable in clinical practice. The assessments will be carried out by trained research assistants or by trained students enrolled in one of the social sciences Master’s program, under supervision of the first author. Figure 2 presents an overview of the measures used for adolescents and parents.

#### Demographics

Demographic data on age, place of birth, and ethnic background will be retrieved from the individual JJI database and from the joint ROM-JJI database. Because these databases do not contain information on family background, housing, past treatment, school careers, and jobs, we will use a short questionnaire to gather these data.

#### Family Interactions

The Family Environment Scale [33] (FES, in Dutch: Gezins Klimaat Schaal, GKS [34]) will be administered to adolescents and parents. This questionnaire consists of the subscales Cohesion, Expressiveness, Conflict, Organization, Control, Moral Standards, and Social Orientation. Each subscale contains 11 items. Questions are answered with “yes” or “no”. The FES has two underlying dimensions, Family Relationship and System Maintenance. The FES has adequate psychometric properties [35]. For example, regarding the internal consistency, the Cronbach alphas for the total group of mothers, fathers, and children differ between .63 (Social Orientation) to .70 (Cohesion). The Cronbach alphas for the System Maintenance and the Family Relationship dimensions are .78 and .82 respectively. The Cronbach alphas for the subgroups are higher than .60 for all subscales, except for Social Orientation for children (alpha=.38) [36].

#### Parenting Stress

We will use the Parenting Stress Questionnaire (PSQ, in Dutch: Opvoedingsbelasting Vragenlijst, OBVL) [37] for assessing the level of parenting stress experienced by parents. The PSQ targets individual characteristics of parents in relation to parenting and to the quality of the parent-child interaction. The questionnaire consists of 34 items to be scored on a four-point scale. Its five subscales are Parent-child relationship problems, Parenting problems, Depressive mood, Parental role restriction, and Physical health problems. The PSQ is shown to be reliable and valid. The Cronbach alphas for the five subscales are .84, .83, .83, .79, and .78 respectively. The total scale was also found reliable (alpha=.90) [38].

#### Satisfaction

We devised a questionnaire based on the Satisfaction Scale [39] and the Client-test (C-test, in Dutch: C-toets [40]), which we will use to determine how satisfied the adolescents and parents are with the JJI. These two questionnaires are shown to be reliable and valid [39,41]. Regarding the Satisfaction Scale for parents, all subscales for the inpatient/residential treatment center population demonstrate good internal consistency, with Cronbach alphas ranging from .76 to .94. For children, all subscales for the inpatient/residential treatment center population show good internal consistency, with Cronbach alphas ranging from .78 to .91, except subscale Access and convenience (alpha=.63) [39]. Cronbach alphas for the four subscales of the parent versions of the Client-test demonstrate good internal consistency, ranging from .77 to .90. The total questionnaire is found to be reliable (alpha=.94). The children version only has a total scale, which is found to be reliable (alpha=.91) [41]. Our satisfaction questionnaire has two parts, part A and part B. Part A contains 14 items to be rated on a three-point scale. It includes items such as “The staff members are friendly”, “I feel that the staff members are interested in me”, “The staff members treat me with respect”, and “The staff members help me dealing with problems”. Part B contains one question, “All things considered, which grade would you give to the service provided by the JJI?”, to be rated on a scale of 1-10.

#### Treatment Motivation

We will apply the Adolescent Treatment Motivation Questionnaire (ATMQ) to measure treatment motivation for adolescents. The ATMQ consists of 11 items to be rated on a three-point scale, adding up to a total score. The construct validity and internal consistency reliability are adequate (alpha=.84) [42]. We added three questions with a three-point scale to the ATMQ about adolescents’ motivation to take part in family therapy during their stay in the short-term stay group and about motivation for continued individual and family therapy after leaving the JJI. We also added four motivation questions to the Satisfaction questionnaire for parents (eg, “I am willing to participate in family therapy during my son’s stay in the JJI”, “I feel that my son needs treatment after his stay in the JJI”).

#### Parents’ Involvement During Their Child’s Detention

To examine to which extent parents are involved with their sons, we will record the number of visits by parents and the purpose of each visit to the JJI. Group workers, team leaders, and psychologists will note when they have had contact via telephone with the parents.

#### Incidents in JJIs

We will gather data on problem behavior as shown by the adolescents from routine daily reports and from JJI database input. JJIs record incidents such as verbal fights, physical fights, quarrels, rule breaking behavior, and possession of contrabands.

#### Cannabis Use

We will gather data on cannabis use from the JJI database. Routinely, JJIs collect a urine sample from the adolescent to check for traces of cannabis use as soon as he is placed in a short-term stay group. Later on during the stay, JJIs regularly perform urine screens, both at scheduled times and at random.

#### JJI Staff

We devised questionnaires for JJI staff (group workers, team leaders, psychologists) about working with families and about using the family perspective in their thinking and in day-to-day interventions. The questionnaire has two parts, part A and part B. Part A contains 12 items to be rated on a five-point scale and includes questions such as “Do you invite parents of every mentor-child for a meeting?”, “Do you invite parents of every mentor-child for a tour through the facility?”, “Do you inform parents on the same day when their child was involved in an incident?”, and “If parents are divorced, do you involve both parents in the same way?”. Part B contains 17 items to be rated on a scale of 1-10. This part includes questions such as “How satisfied are you with the course of the contact with the parents?”, “How satisfied are you with the way in which you involve parents during their son’s stay?”, and it includes statements such as “Parents are difficult to work with”, “Parents are indispensable for reducing recidivism”, and “Parents are a source of support for staff”.

These questionnaires will be filled out every three months. On an additional form, psychologists will note where the adolescent is going to live after leaving the short-term stay group.

To assess if staff members adhere to the guidelines of the FC program, they will use logbooks and will fill out short forms on family-centered activities undertaken. This will enable us to assess program integrity. The overviews of these logs are shared during team meetings and with the managements, enabling managers and team leaders to monitor and direct the organization of family-centered activities.

### Procedure and Instruments of the Qualitative Part of the Study

Before the interview, the participant will complete a short demographic questionnaire. The interview will be about 60 to 90 minutes and will be audio recorded. The recording will be stopped during the interview if so requested by the participant. The semi-structured interviews will be conducted by qualified trained students enrolled in the last year of either a Bachelor’s or a Master’s program of Social Work or another social science.

The interviews are structured using a topic list [43]. We drafted a topic list for each group of participants (adolescents, parents, FC group workers, and UC group workers). The topic lists were devised following deductive and inductive strategies. Deductively, topics were derived from a review of literature of factors that contribute to the success of family-centered work in institutions similar to JJIs. Inductively, experiences from group workers, parents, and adolescents were used to supplement the topic list. Additionally, each interview can influence the construction of the topic list as new themes may arise. The themes of the final topic lists are represented by questions and are displayed in Table 1 and Table 2. Although the topics follow a logical order in themes, the topic lists will be used in the order as the interviewer sees appropriate, based on the answers of the respondents. Based on further subtopics and keywords the interviewer will probe for more information on each main theme as specified in Tables 1 and 2.

**Table 1:** Main themes of the topic lists for interviewing adolescents and parents.

Adolescents and parents	Adolescents only	Parents only
To what extent are parents currently involved?	Do you consider the involvement of your parents as being important?	To what extent and in which way do you wish to be involved?
How can parents be motivated for involvement?	How should the JJI involve parents?	
What are your expectations of staff in involvement and contact?	How can the JJI motivate adolescents for FC?	
Which factors influence involvement and in which ways?	Which reasons do adolescents have to object to FC?	
How can we explain the surprising preliminary finding in the quantitative pilot stage that parents and youths report few family problems while they also report to be motivated for family therapy?		

**Table 2:** Main themes of the topic lists for interviewing group workers.

Group workers FC and UC	Group workers FC only	Group workers UC only
How do you feel about the involvement of parents?	What is Family-centered Care?	What is parental participation?
What do you think about the following elements in parental participation: knowing, discussing, activities, and deciding?	Which changes in practice did you notice since the implementation of FC?	What do you expect of FC when it will be implemented in your group in the future?
How is the atmosphere in your team?	How has FC been implemented in your team?	Which changes are necessary before your team is ready for the implementation of FC?
What is your role within your team?	How do you feel about the FC training?	
Do you have sufficient skills for involving parents?		
Do your colleagues have sufficient skills for involving parents?		
To what extent do managers support you in involving parents?		
What pros and cons of FC do you see?		
Do you have tips for involving parents?		

### Analyses

#### Quantitative Analyses

All statistical analyses will be performed using SPSS 23. In a future paper, we will provide a flowchart of participants in our study, including reasons for exclusion. Descriptive statistics will be presented as means and standard deviations for all continuous variables and subscales. Additionally, frequency distributions or qualitative descriptions of all categorical variables will be presented for each group. The groups will be defined as FC or UC. We will test if these groups differ on demographic factors. If these differences exist, we will use these factors as covariates in our analyses. If necessary, we will also include the JJI in which an adolescent is placed as a covariate. We will perform within-group pre-post comparisons, between-group comparisons (FC vs UC), and repeated measures analyses. The selection of a specific test will depend on which hypothesis is tested and on the characteristics of the corresponding data (eg, categorical, ordinal, or interval level and normally or non-normally distributed). Table 3 shows the planned analyses to test our hypotheses for comparing FC with UC in case of normally distributed data. For combining the within-group pre-post comparisons and the between-group comparisons in our analyses, we will use the repeated measures ANOVA. Because the normality of the distribution of the data cannot be determined beforehand, the final analyses will be selected after the data is gathered. In analyzing the hypotheses, two-tailed analyses will be performed and we will correct for multiple testing.

**Table 3:** Planned analysis for between-group hypotheses.

Hypothesis	Data source	Analysis
FC increases parents’ involvement with their detained child	Registration logs visits	Unpaired *t* test
FC increases the motivation of the adolescents and parents for accepting treatment and guidance by JJI staff and for taking part in family meetings	ATMQ youth total score Motivation items youth	Unpaired *t* test Pearson’s Chi-square test
	Motivation items parents	Pearson’s Chi-square test
FC adolescents show less problem behavior	Incidents in JJI Cannabis database	Unpaired *t* test Unpaired *t* test
FC improves family interactions	FES	Unpaired *t* test
FC parents experience less parenting stress	PSQ	Pearson’s Chi-square test
FC youth more often return to their families’ home upon discharge	Registrations logs living situation after discharge	Pearson’s Chi-square test
FC enhances adolescents’ and parents’ satisfaction with the JJI	Satisfaction questionnaire-A Satisfaction questionnaire-B	Pearson’s Chi-square test Unpaired *t* test
In FC groups, JJI staff members are more satisfied, feel more confident in their contact with parents, and more often incorporate the family perspective in thinking	Questionnaire staff-A	Generalized estimating equations
	Questionnaire staff-B	General linear model repeated measures

#### Qualitative Analyses

The recordings of the interviews will be transcribed verbatim and imported into ATLAS.ti, a computer program facilitating the analysis of qualitative data. The students will be trained to code the data using a code tree representing the topic list. This first draft of the deductively developed code tree will be complemented with codes inductively derived during the coding process, as new themes will appear in the answers of participants [44]. The first author and the students will work in a cyclic process. This first phase of open coding will be followed by a second phase of axial coding. During axial coding, codes will be further interpreted and reorganized based on the interview fragments they refer to. Codes can get split, merged, and joined into more abstract central themes. Code families will be constructed enabling further analysis of the data. The third and last phase of the analytic process, selective coding, will enable theoretical interpretations aimed at finding more general patterns [43]. Finally, this analytic process enables us to explain the underlying mechanisms influencing parental involvement during their child’s detention.

### Ethics

The medical ethical board of the Leiden University Medical Center reviewed our study. The board ruled that our study falls outside the realm of the WMO (Dutch Medical Research in Human Subjects Act) and that it conforms to Dutch law, including ethical standards.

## Discussion

Until recently, care for adolescents detained in a juvenile justice institution (JJI) has been mainly youth-centered with interventions targeting a youth’s problem behavior without much regard for the youth’s social environment, in particular the family. The Dutch government and the JJIs are convinced that outcomes for detained adolescents are more improved if their parents are allowed to meet and to talk with their child more often, to have direct and extensive contact with JJI staff, to join parent meetings organized by the JJI, and to have a say in decisions regarding their child. As research supports these notions [3,8-10,13,45], this calls for drastically revising current JJI programs [12,18,19]. Two JJIs in the Netherlands combined efforts with universities, colleges, and mental health centers within the Academic Workplace Forensic Care for Youth (AWFZJ) to introduce family-oriented care in their institutions. The AWFZJ developed two programs for family-centered care (FC), for youths detained in groups for short-term and long-term stay, respectively. In FC, staff members receive training, ongoing coaching, and yearly booster sessions on working with parents. The current paper reports on the design of a study evaluating FC in the first two years after the first steps of its implementation in short-term stay groups. After the pilot stage in 2012, the second stage of the study started in 2013 and we completed the data collection procedures in 2015. Currently, we are analyzing the first sets of outcomes and we expect to report on them over the coming years.

Our study has an explanatory sequential mixed methods design, combining quantitative and qualitative approaches in a practice-based study. In order to overcome the challenge of conducting practice-based research with possible tension between practice and science [25,46], we established good working relationships with the staff, collaborating with the same goal in mind: evaluating and eventually improving FC. Over the course of our study, we kept in mind the need to be flexible in carrying out practice-based research [25], possibly resulting in changes in practical ways of collecting data while adhering to our study’s methods.

During our study, we undertook a few actions as discussed in the Methods section to ensure that staff members benefit from our study. First, we discussed our research design in a workgroup with staff in each institution. We enabled staff members to provide feedback on our original design and we incorporated their suggestions in our final study. The workgroups supported our study by serving as a bridge between practice and science. Second, we helped scheduling the assessments and interpreting the scores so that they were usable in clinical practice. Third, we provided feedback on the registered information of staff’s family-oriented actions during team meetings and to the managements of the two JJIs. Using research information as feedback for practice helps staff members to understand the benefits of conducting research. While our study is useful for practice, this advantage also has a down side. Along the course of our study, practice can evolve as staff might improve in the way of working with parents. Nevertheless, by directly using results of our study in practice, we meet an important requirement of practice-based research [25,47].

Close collaboration with the JJI managements is necessary to overcome possible bottlenecks during our practice-based study. Since the wish to develop and evaluate FC originates from the institutions themselves, the joint goal to improve parental participation is emphasized. JJIs are also interested in more distal outcomes such as recidivism rates. We recognize the importance of studying the long-term effects of implementing FC and therefore suggest future research to incorporate distal outcomes.

In conclusion, we expect the results of our study to contribute to practice by showing how to organize FC and by providing suggestions for improving the FC program, which consequently can lead to improved care for detained adolescents and their families.

## Abbreviations

AWFZJ: Academic Workplace Forensic Care for Youth
ATMQ: Adolescent Treatment Motivation Questionnaire
FC: family-centered care
FES: Family Environment Scale
FFT: functional family therapy
GKS: Gezins Klimaat Schaal
JJI: juvenile justice institute
MDFT: multidimensional family therapy
OBVL: Opvoedingsbelasting Vragenlijst
PIJ: Placement in an Institution for Juveniles for mandatory treatment
PSQ: Parenting Stress Questionnaire
ROM: Routine Outcome Monitoring
UC: usual care
WMO: Dutch Medical Research in Human Subjects Act
